# High Mineralization Capacity of IDG-SW3 Cells in 3D Collagen Hydrogel for Bone Healing in Estrogen-Deficient Mice

**DOI:** 10.3389/fbioe.2020.00864

**Published:** 2020-08-31

**Authors:** Kaizhe Chen, Qi Zhou, Hui Kang, Yufei Yan, Niandong Qian, Changwei Li, Fei Wang, Kai Yang, Lianfu Deng, Jin Qi

**Affiliations:** ^1^Shanghai Key Laboratory for Prevention and Treatment of Bone and Joint Diseases, Shanghai Institute of Traumatology and Orthopaedics, Ruijin Hospital, Shanghai Jiao Tong University School of Medicine, Shanghai, China; ^2^Department of Orthopedics, Shanghai Tenth People’s Hospital, Tongji University School of Medicine, Shanghai, China; ^3^Department of Orthopedic Surgery, Shanghai Jiao Tong University Affiliated Sixth People’s Hospital, Shanghai, China

**Keywords:** tissue regeneration, BMSCs, osteoblast, bone, osteocyte

## Abstract

Tissue engineering with 3D scaffold is a simple and effective method for bone healing after large-scale bone loss. So far, bone marrow-derived mesenchymal stem cells (BMSCs) are mostly used in the treatment of bone healing in animal models due to their self-renewal capability and osteogenic potential. Due to the fact that the main functional cells in promoting osteoid mineralization and bone remodeling were osteocytes, we chose an osteoblast-to-osteocyte transition cell line, IDG-SW3, which are not proliferative under physiological conditions, and compared the healing capability of these cells to that of BMSCs in bone defect. *In vitro*, IDG-SW3 cells revealed a stronger mineralization capacity when grown in 3D collagen gel, compared to that of BMSCs. Although both BMSC and IDG-SW3 can generate stable calcium-phosphate crystal similar to hydroxyapatite (HA), the content was much more enriched in IDG-SW3-mixed collagen gel. Moreover, the osteoclasts co-cultured with IDG-SW3-mixed collagen gel were easier to be activated, indicating that the IDG-SW3 grafting could promote the bone remodeling more efficiently *in vivo*. Last, in order to reduce the self-healing capability, we assessed the healing capability between the IDG-SW3 cells and BMSCs in osteoporotic mice. We found that the collagen hydrogel mixed with IDG-SW3 cells has a better healing pattern than what was seen in hydrogel mixed with BMSCs. Therefore, these results demonstrated that by promoting osteoblast-to-osteocyte transition, the therapeutic effect of BMSCs in bone defect repair could be improved.

## Introduction

Bone healing after large-scale bone loss remains a major clinical challenge. Tissue engineering is a simple and effective method for generating solid tissues, and it can be used to produce implantable bone grafts for reconstructive surgery and has great prospects in large-scale bone loss repair (El-Ghannam, 2005; Freitas et al., 2019). Three-dimensionally cultured osteogenic cells, such as osteoblasts or bone marrow-derived mesenchymal stem cells (BMSCs) in porous scaffolds, are in a favorable environment for the subsequent regeneration of bone tissue under the required conditions (Yoshikawa and Myoui, 2005; Yuan et al., 2007). So far, BMSCs are mostly used in the treatment of bone healing in animal models due to their self-renewal capability and osteogenic potential (Zong et al., 2010; Koob et al., 2011).

Bone healing is composed of a well-characterized cascade of events (Einhorn, 2005). At first, bone defects recruit a large number of inflammatory cells and osteoprogenitor cells to the local site to promote hematoma formation. Second, BMSC-derived osteogenic cells generate a type I collagen-rich osteoid matrix to form a soft mineralization callus. Then, the embedded osteoblasts differentiate into mature osteocytes and gradually mineralize the collagen fibers in the osteoid space with calcium nodules to establish woven bone (Barragan-Adjemian et al., 2006). Finally, the woven bone experiences a bone remodeling process to lamellar bone and restores the medullary cavity. Obviously, the spatial and temporal coordination of different osteogenesis-related functions through the healing process determines the healing efficiency. Among them, osteoid mineralization and bone remodeling are actually functions of osteocytes. In addition, the extensive lacunae–canaliculi network formed by osteocytes also functions in bone remodeling and mineral deposition (Feng et al., 2006; Bonewald, 2011; Dallas et al., 2013).

IDG-SW3 is a cell line of osteoblasts differentiating into osteocytes and undergoes transitions through stages of being osteoblasts, preosteocytes, and finally osteocytes. We found that IDG-SW3 have more osteogenic capacity when grown in three-dimensional culture compared to BMSCs *in vitro*. We observed a more extensive dendrite network formed in the 3D culture system mixed with IDG-SW3 cells compared to that of the BMSCs. Moreover, the functional proteins expressed in IDG-SW3 cells at different stages perfectly met the functional requirements of relative stages during bone formation and bone remodeling. Although both BMSC and IDG-SW3 can generate stable hydroxyapatite (HA), IDG-SW3 cells revealed a stronger mineralization capacity, and the gel mineralized by IDG-SW3 revealed a superior elastic modulus. Importantly, we also found that mature IDG-SW3 cells promote osteoclast activation. Last, IDG-SW3 cells mixed with collagen hydrogel has a better effect in promoting osteoporotic bone healing than what was seen in BMSCs.

## Materials and Methods

### Cell Culture

Mouse BMSCs were extracted from the femurs of three 8-week-old male mice. The isolated bone marrow was pooled together and cultured with α-MEM medium (Minimum Essential Medium with ribonucleosides and deoxyribonucleosides, Gibco, #32571-036) with 10% FBS and 1% penicillin/streptomycin. After 3 days, the non-adherent cells were removed and the fourth passage of the adherent cells was used for subsequent experiments.

IDG-SW3 cells, a gift from Dr. Lynda Bonewald (Indiana University), were proliferated on collagen-coated plates in α-MEM medium supplemented with 10% FBS, 50 U/ml of IFN-γ, and 1% penicillin/streptomycin at 33°C and 5% CO_2_.

The 1 × 10^5^ IDG-SW3 cells or BMSCs were mixed with 1-ml mixture of type I Rat tail collagen gel (Gibco, #A10483-01) and matrigel matrix (Corning, #356238), according to the manufacturer’s protocol. For osteogenic differentiation, the gels were cultured with osteogenic medium (α-MEM medium supplemented with 10% FBS, 50 μg/ml of ascorbic acid, 4 mM β-glycerophosphate, and 1% penicillin/streptomycin) at 37°C with 5% CO_2_, and the medium was replaced every other day.

### Cytoskeleton Staining

After culturing with osteogenic medium for 1 week, collagen gels containing BMSCs or IDG-SW3 cells were fixed with 4% paraformaldehyde for 10 min and washed three times with PBS. Then, the cytoskeletons were labeled with 0.5 ml of 5 μg/ml Alexa Fluor 594 phalloidin (Life Tech, United States) for 30 min at room temperature. High-resolution imaging of Z-stack of labeled cells in the gel was observed by LSM800 laser scanning confocal microscopy (ZEISS, Germany). The dendrite network was reconstructed in IMARIS software with *Filaments* module (Version 11, Bitplane, Switzerland). The parameters of the *Filaments*, including dendrite volume, dendrite number, and dendrite length, were both automatically exported with IMARIS software.

### Quantitative Real-Time PCR Analysis

The collagen gels containing 1 × 10^5^ BMSCs or IDG-SW3 cells were cultured in osteogenic medium. At different time points (0, 2, 4, and 6 weeks), total RNA was extracted from gels using the Trizol reagent (Invitrogen, United States) according to the manufacturer’s instructions. Complementary DNA was synthesized from 1 μg of total RNA using PrimeScript RT reagent Kit (TakaRa, Japan) following the manufacturer’s protocol. After reverse transcription reaction, real-time PCR was performed using a SYBR Green qRT-PCR kit (TakaRa, Japan) and an ABI 4000 Real-Time PCR System (Applied Biosystems, United States). β-actin was used as the housekeeping gene, and all reactions were run in triplicate. The mouse primer sequences for *Alp* (Accession Number: NM_001287172.1), *Oc* (Accession Number: NM_001032298.3), *Pdpn* (Accession Number: NM_010329.3), *Dmp-1* (Accession Number: NM_016779.2), *Fgf23* (Accession Number: NM_022657.4), *Sost* (Accession Number: NM_024449.6), and β-*actin* (Accession Number: NM_007393) are available in Supplementary Table S1.

### Western Blot Analysis

The collagen gels containing 1 × 10^5^ BMSCs or IDG-SW3 cells were cultured in osteogenic medium. Then, the gels were subjected to 4 × SDS lysis buffer at the indicated time. After sonication, samples were subjected to SDS electrophoresis and transferred to a polyvinylidene difluoride membrane (0.22 μm, Millipore, United States). The membranes were first blocked with 5% skimmed milk in TBS-T for 1 h and then incubated at 4°C overnight with primary antibodies as follows: DMP-1 (#ab103203), RANKL (#ab45039), and β-actin (#ab179467). After being washed three times with TBS-T, the membranes were incubated with HRP-conjugated secondary antibodies (1:5000, Jackson ImmunoResearch, United States). The images were visualized with enhanced chemiluminescence detection system as recommended by the manufacturer. The study was performed in triplicate.

### Alizarin Red S Staining

Alizarin Red S staining was used to highlight the mineralization nodules formed in collagen gels. The cells were cultured in osteogenic medium. After 4 weeks, gels were fixed with 4% paraformaldehyde and washed three times with PBS. The fixed gels were further washed with distillated water in order to remove any salt residues and then a solution of 2% (wt/v) Alizarin Red S (ARS, Sigma, United States) was added so that it covered the entire surface of the gels. After an incubation of 10 min at room temperature, the excess of ARS was washed with distillated water. The images were acquired with a stereomicroscope or an LSM800 laser scanning confocal microscope (ZEISS, Germany). The study was performed in triplicate.

### Scanning Electron Microscopy (SEM)

For scanning electron microscopy, the mineralized gels were lyophilized, cut into thin slices, and then coated with a 10-nm gold layer and observed in Hitachi S-4700 at an accelerating voltage of 10 kV. Energy-dispersive X-ray (EDX) was performed with an energy spectrum detection range of B5-U92 at an accelerating voltage of 15 kV to chemically characterize the mineralized gels. Average Ca/P ratios were determined from triplicate measurements.

### X-Ray Diffraction (XRD)

The composition of the samples was determined by XRD based on the International Diffraction Data Center database. A Shimadzu XRD-6000 diffractometer was used to obtain XRD characterization data. The mineralized gels were freeze-dried first and then were pressed into the cover glass etched area for testing. During the experiment, a Ni filter and a copper excitation emission line (CuKα, 1.5406 Å, 40 kV, 40 mA) were used. Profiles were obtained between 5° and 70°, and a count of 5 s was performed at room temperature every 0.013°.

### Fourier Transform Infrared Spectra (FTIR)

To assess the composition of the deposited minerals in the collagen gel, FTIR spectra were performed using the freeze-dried gel on a Nicolet 6700 spectrometer (Thermo Scientific) with iTR mode. Spectra were acquired over a spectral range of 400–4000 cm^–1^ at a spectral resolution of 2 cm^–1^ and 64 scans.

### Viscoelastic Properties of Mineralized 3D Gel

Viscoelastic properties were determined by using a rheometer (TA Instruments, Model AR2000ex) equipped with 40-mm-diameter stainless steel parallel plate geometry. The mineralized gels were freshly prepared before rheological tests. The operation temperature was maintained at 37°C. Storage modulus (*G*′) and viscous modulus (*G*″) were measured by performing frequency sweeps between 0.01 and 1.0 Hz.

### Bone Marrow-Derived Macrophage (BMM) Isolation and Osteoclast Co-culture

Primary BMMs were isolated from the long bones of 8-week-old C57BL/6J mice. Cells were isolated from the femur and tibiae bone marrow and cultured in a 100-mm dish with complete α-MEM medium for 24 h. Non-adherent cells were harvested and cultured with fresh medium containing 50 ng/ml M-CSF. Three days later, the adherent cells were harvested as osteoclast precursors (pre-osteoclasts). These cells were then seeded on the collagen gels containing BMSCs or IDG-SW3 cells after 4 weeks of differentiation, and further co-cultured with α-MEM medium containing 10% FBS, 1% penicillin/streptomycin, 10 nM 1,25-dihydroxyvitamin D3, and 1 μM PGE2 for an additional 7 days. Cell culture medium was half-replaced every 2 days. Next, gels were washed twice by PBS and fixed with 4% paraformaldehyde for 15 min and subjected to tartrate-resistant acid phosphatase (TRAP) staining using the manufacturer’s protocol (Sigma, #387A-1KT, United States). TRAP-positive area was visualized with a stereomicroscope. The TRAP activity was analyzed by a tartrate-resistant acid phosphatase assay kit (#P0332, Beyotime, China).

### Animal Experiment

Forty 8-week-old C57BL/6 female mice were purchased from Shanghai SLAC Laboratory (Shanghai, China). 0.25% pentobarbital sodium dissolved in saline were i.p. injected for anesthesia, according to body weight of each mouse. After anesthesia, four of them were subjected to sham-operated experiment (as SHAM group), where the rest of them were all subjected to bilateral ovariectomy, as OVX mice.

After 2 weeks, the OVX mice were randomly divided into three groups and all were subjected to drill-hole defect surgery in the right femur. A skin incision on the outer side of the right femur was made and a blunt dissection of the quadriceps was performed to expose the femoral diaphysis. Perforations were then locally generated through the posterior and anterior cortices using a Kirschner wire. The 0.7-mm diameter of the Kirschner wire was measured by Vernier calipers at the thickest place (Supplementary Figure S1A). After rinsing by injection of saline to remove bone fragments from the cavity, the holes were filled with collagen I gels only, or collagen I gels containing BMSCs or IDG-SW3 cells, as control group, BMSC group, and IDG-SW3 group, respectively. Then, the muscles were subsequently repositioned, and the skin was closed with suture.

At 7 days after drill surgery, the SHAM mice and five of the OVX mice in each group were sacrificed and the femora were subjected to radiographic analysis. At 14 days after drill surgery, the rest of the mice were all sacrificed and the right femora were subjected to radiographic and histological analysis.

All the procedures involving mice experiments were approved by the Shanghai Jiao Tong University Animal Care and Use Committee and in direct accordance with the Ministry of Science and Technology of the People’s Republic of China on Animal Care guidelines.

### Radiographic Examination

At day 7 after drill-hole surgery, the five OVX mice from each group and the SHAM mice were sacrificed. The tibiae of SHAM mice (*n* = 4) and OVX mice (*n* = 5) from the hydrogel group were subjected to pQCT equipment (XCT 540; Stratec, Birkenfeld, Germany) analysis for osteoporosis identification. For the tibiae, 2 cross-sections (at 0.25-mm intervals) were analyzed for bone mineral density (BMD), 1.8 mm distance from growth plate of the tibia, including trabecular and cortical bone. Standardized analysis with an image voxel size of 0.07 mm was performed. The right femora of the five OVX mice from each group were subjected to X-ray scanning for mineralization callus formation.

At day 14, after drill-hole surgery, the rest of the mice were sacrificed and the right femora were subjected to μ-CT analysis. The trabecular ROI including defect area over 1.5 mm was used for trabecular bone parameters analysis. The BV/TV and Tb.Sp of the defect area were used for bone healing measurement with CTAn. Three-dimensional images of the local defect area were reconstructed in CTVox with two-dimensional images. μ-CT analysis was performed on Skyscan 1172 (Aartselaar, Belgium) with a 10-μm isotropic voxel size, 50 keV, 500 μA, and 0.7° rotation step, in accordance with the recommendations of the American Society for Bone and Mineral Research (ASBMR) (Dempster et al., 2013).

### Histological Analysis

After μ-CT scanning, the right femurs were decalcified in 10% EDTA for 3 weeks and then dehydrated and embedded in paraffin. Histological sections (5 μm thick) were prepared for H&E and TRAP staining. The specimens were then examined and photographed under a high-quality upright microscope (ZEISS, Germany).

### Statistical Analysis

All of the original data were analyzed and plotted with GraphPad Prism software (version 7.0c). Statistical analysis was performed with two-tailed unpaired *t* test or ordinary one-way ANOVA analysis. Data were all shown as means ± s.e.m. *P* values indicated the difference significance.

## Results

### Extensive Dendrite Network of IDG-SW3 Cells Cultured in Collagen Gel

**FIGURE 1:**
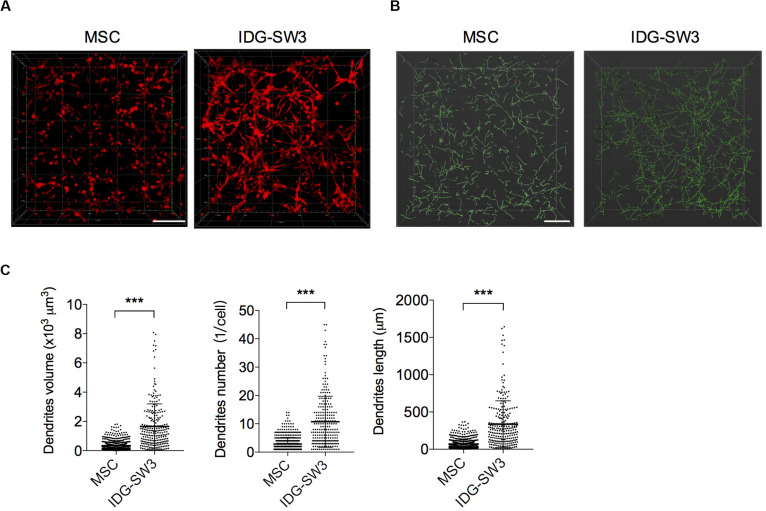
Extensive dendrite network of IDG-SW3 cells cultured in collagen gel. **(A)** Representative images of cytoskeleton, stained by phalloidin from IDG-SW3 cells and BMSCs cultured in 3D type I collagen gel with osteogenic medium after 7 days. **(B)** Dendrite network modeling of IDG-SW3 cells and BMSCs, analyzed by *Filaments* module of IMARIS software with the cytoskeleton images **(A)** as original data. **(C)** Parameters of the dendrite network analyzed by IMARIS software and shown with scatter plots. Bars, 200 μm. Data were represented as means ± s.e.m. ***P < 0.001.

As noted, it is difficult to induce the formation of an intact dendrite network with conventional 2D *in vitro* culture conditions. Thus, to better mimic the *in vivo* physiological circumstances and therefore facilitate the differentiation of osteogenic cells, BMSCs and IDG-SW3 cells were cultured within a 3D type I collagen gel matrix environment to keep their 3D ellipsoid morphologic cell shapes intact. According to the expression pattern of osteoblastic- and osteocytic-specific markers in IDG-SW3 cells over time, as summarized by Woo et al. (2011) 7-day osteogenic media culturing causes IDG-SW3 cells to differentiate into preosteocytes, at which point dendrite formation and elongation are mainly promoted by E11 protein. Therefore, we stained the cytoskeletons with phalloidin to analyze the dendrite network formed in collagen gel. Representative confocal Z-stack images (Figure 1A) showed a well-established dendrite network in IDG-SW3 cells cultured in collagen gel. To compare the parameters of the dendrite network formed by these two cell lines, we loaded these 3D images into IMARIS software and reconstructed the dendrite network with *Filaments* module (Figure 1B). Statistical data showed that the space occupied by the dendrite network, the number of dendrites per cell, and the dendrite length per cell were all increased in IDG-SW3 cells grown in collagen gel (Figure 1C). Thus, the dendrite network formed by IDG-SW3 cells in collagen gel was more extensive than that formed by BMSCs.

### Spatiotemporally Proteomic Changes of IDG-SW3 Cells Cultured in Collagen Gel

**FIGURE 2:**
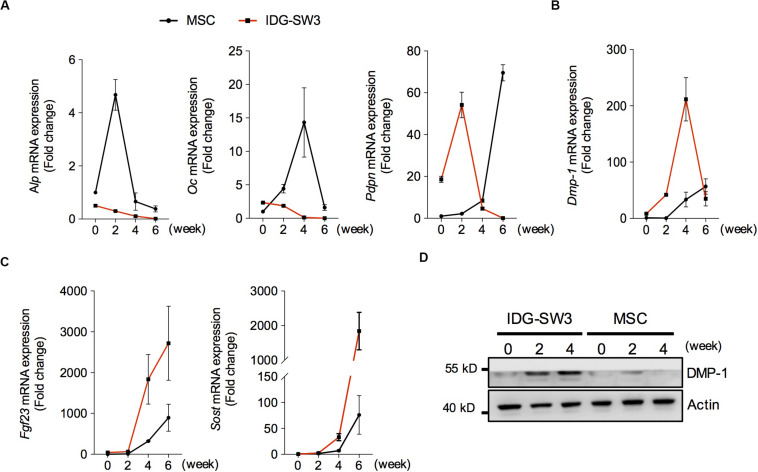
Spatiotemporally proteomic changes of IDG-SW3 cells cultured in collagen gel over time. **(A–C)** Relative changes of mRNA level of osteoblastic and osteocytic markers expressed in IDG-SW3 cells and BMSCs over differentiating time. **(D)** The protein level of osteocytic markers expressed in IDG-SW3 cells and BMSCs over differentiating time. Data were represented as means ± s.e.m.

To investigate the osteogenic differentiation of these two cell lines in collagen gel, we examined the mRNA levels of osteoblastic- and osteocytic-specific markers over time. At the 2nd week, the increased alkaline phosphatase (ALP) and osteocalcin (OC) in the BMSC-mixed collagen gel indicated that the BMSCs differentiated into osteoblasts within 2 weeks (Figure 2A). Then, the decreased ALP and increased preosteocyte marker E11 suggested that preosteocytes had formed at the 4th week (Figure 2A). The highly expressed E11 and dentin matrix protein 1 (DMP-1) were maintained in BMSC-mixed collagen gel until the 6th week (Figures 2A,B), indicating that these cells have not yet differentiated into fully functional, mature osteocytes, although fibroblast growth factor 23 (FGF23) and sclerostin could be detected in these cells (Figure 2C). In contrast, OC protein in IDG-SW3 cells was highly expressed at the beginning of differentiation, and it decreased thereafter (Figure 2A). At the 2nd week, the peaked E11 expression and decreased ALP or OC expression indicated that IDG-SW3 cells had differentiated into preosteocytes, which was accomplished 2 weeks earlier than It was with the BMSCs (Figure 2A). At the 4th week, the peak DMP-1 expression began to decrease and was replaced by the high expression of FGF23 and sclerostin proteins, indicating that fully functional, mature osteocytes were formed. Consistently, Western blot experiments also showed that the protein level of DMP-1 in IDG-SW3 cells was gradually increased in the 4 weeks, whereas in BMSCs, only few expressions of DMP-1 protein was observed (Figure 2D). These results demonstrated that IDG-SW3 cells differentiated into mature osteocytes earlier than BMSCs.

### Enhanced Mineralization Activity of IDG-SW3 Cells Than BMSCs

**FIGURE 3:**
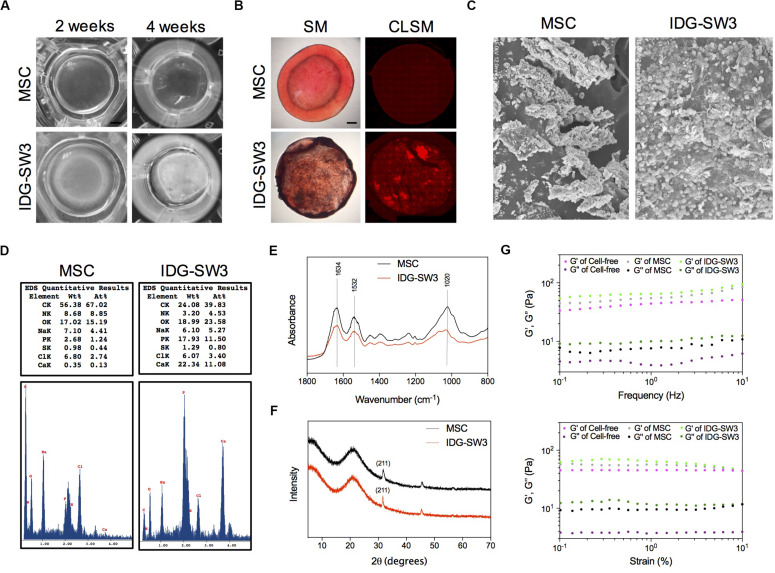
Enhanced mineralization activity of IDG-SW3 cells compared to BMSCs. The physical and chemical properties of mineralized 3D gels containing IDG-SW3 cells and BMSCs after 4-week differentiation. **(A)** The outward appearance of the mineralized 3D gels. Bar, 2 mm. **(B)** Alizarin Red S staining of the mineralized 3D gels, visualized with a stereomicroscope (SM) or a confocal laser microscope (CLSM). Bar, 2 mm. **(C)** Scanning electron microscope images of the mineralized 3D gels surface. **(D)** Energy-dispersive X-ray analysis of the mineralized 3D gels. **(E)** Fourier transform infrared spectra analysis of the mineralized 3D gels. **(F)** X-ray diffraction analysis of the mineralized 3D gels. **(G)** Viscoelastic properties of the 3D gels with or without cells.

Next, we analyzed the physical and chemical properties of the mineralized 3D gels containing IDG-SW3 cells and BMSCs during differentiation. First, the 3D gel containing IDG-SW3 cells became gradually opaque over time until the 4th week, while the 3D gel containing BMSCs was still transparent (Figure 3A). Alizarin red staining confirmed the presence of large amounts of calcium in the IDG-SW3-mixed gel but very little calcium in the BMSC-mixed gel (Figure 3B). To visualize the substances formed in the gels more clearly, scanning electron microscope was employed; the results revealed that there were a large number of mineralized particles widely distributed in the IDG-SW3-mixed gel, whereas the gel containing BMSCs has fewer mineralized particles located near the cells (Figure 3C).

Then, we analyzed the chemical properties of these mineralized collagen gels with EDX, Fourier transform infrared spectra (FTIR), and XRD analysis. EDX results showed that the weight of calcium and phosphorus in the collagen gel mixed with IDG-SW3 cells was much higher than that in the gels mixed with BMSCs (22.34 to 0.35% calcium and 17.93 to 2.68% phosphorus, respectively) (Figure 3D). The FTIR data showed a comparable spectra between IDG-SW3- and BMSCs-mixed gel, with three peaks at 1634, 1532, and 1020 cm^–1^, in which the 1020 cm^–1^ peak indicates the poorly crystalline apatite presented at the early stage of mineralization (Rey et al., 1991), whereas the 1634 cm^–1^ and 1532 cm^–1^ peaks were attributable to proteins and amino acids, respectively (Figure 3E). Similar to the results, the XRD patterns of the collagen gels both have a 2*θ* halo at 31.9° (Figure 3F), which corresponds to one of the diffraction planes (211) of the HA crystallite (Venkatesan and Kim, 2010).

Finally, rheological studies were carried out to characterize the physical properties of these gels. At first, the storage modulus (*G*′) and viscous modulus (*G*″) in the gels mixed with cells were both increased compared to cell-free gels. Moreover, these two parameters were obviously enhanced in the IDG-SW3-containing collagen gel compared with that of the BMSC-containing collagen gel (Figure 3G). These results demonstrated that the mineralized particles formed in both gels were similar to the hydroxyapatite, but the content were much more enriched in IDG-SW3-mixed collagen gel, as well as the rheological properties.

### IDG-SW3 Cells Promote Osteoclast Activation and RANKL Expression

**FIGURE 4:**
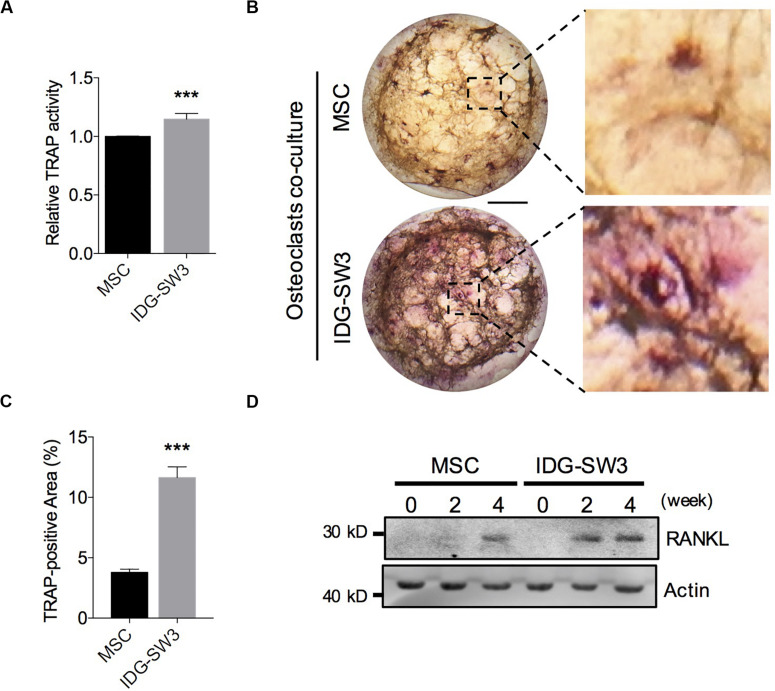
IDG-SW3 cells promote osteoclast activation and RANKL expression. Relative TRAP activity **(A)**, representative TRAP staining images **(B)**, and TRAP-positive area analysis **(C)** of the osteoclasts co-cultured on 3D gels containing IDG-SW3 cells or BMSCs. **(D)** The content of RANKL protein expressed in 3D gels containing IDG-SW3 cells or BMSCs over differentiating time. Bar, 2 mm. Data were represented as means ± s.e.m. ***P < 0.001.

Osteoclasts (OCs) are responsible for degrading the bone matrix, thereby initiating bone remodeling and maintaining the integrity of the bone tissue, under control of receptor activator of nuclear factor-κ B ligand (RANKL) stimulation (Charles and Aliprantis, 2014). Recent studies have indicated that the RANKL protein for osteoclastogenesis *in vivo* mainly comes from the osteocytes (Nakashima et al., 2011). In the later stages of bone healing, an extensive lacunar–canaliculi network ensures osteoclast activation and rapid bone remodeling (Bonewald, 2011; Dallas et al., 2013). Therefore, we further investigated the capability of these two cells in promoting osteoclast activation. We co-cultured bone marrow-derived macrophages on the upper layer of the collagen gel, which contained IDG-SW3 cells or BMSCs that had already differentiated for 4 weeks. After 5 days, the monocytes were tested for TRAP activity and TRAP staining. The results showed that monocytes co-cultured with IDG-SW3 cells had higher TRAP activity than BMSCs (Figure 4A). Similar to the results, the TRAP-positive areas were more extensive in the IDG-SW3 group (Figures 4B,C). Notably, we detected RANKL content in both gels, whereas the gel containing IDG-SW3 cells had more RANKL protein than the gel with BMSCs (Figure 4D); this might be due to the more fully functional osteocytes that were generated in the gel containing IDG-SW3 cells. Therefore, we believe that IDG-SW3 cells could more rapidly repair bone defects than BMSCs.

### Improved Drill-Hole Bone Healing in Estrogen-Deficient Mice With IDG-SW3 Cells

In order to observe the difference capability in bone formation between BMSCs and IDG-SW3 *in vivo*, we selected a drill-hole cortical bone healing model (He et al., 2011). At the same time, in order to reduce the self-healing capability in normal mice, we observed the osteogenic differences between the two cells in osteoporotic mice, in which the osteogenesis and bone remodeling capability were both impaired (He et al., 2011). Two weeks after ovariectomy (OVX), a 0.7-mm hole was drilled at the metaphysis of the right femora through the posterior to anterior cortex, and then it was filled with collagen I gel containing 1 × 10^5^ BMSCs or IDG-SW3 cells. We also filled it with hydrogel only as a cell-free group (Supplementary Figure S1A).

**FIGURE 5:**
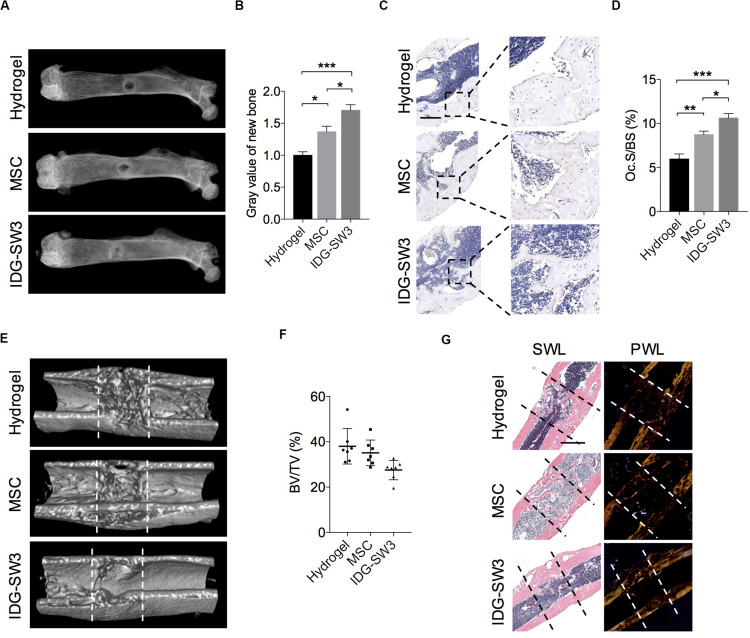
Improved drill-hole bone healing in estrogen-deficient mice with IDG-SW3 cells. **(A–D)** After drill-hole surgery for 1 week, mice were sacrificed and the right femora were subjected to radiographic and histological examination (*n* = 5). **(A)** Representative X-ray images. **(B)** Grayscale analysis of the new bone formed in defect area. **(C)** Representative TRAP staining images of the defect area. Bar, 200 μm. **(D)** Quantification of the TRAP staining by osteoclast surface per bone surface (Oc.S/BS). **(E–G)** After drill-hole surgery for 2 weeks, mice were sacrificed and the right femora were subjected to radiographic and histological examination (*n* = 7). **(E)** 3D μ-CT images of the defect area. **(F)** μ-CT analysis of bone volume per tissue volume (BV/TV) in the intramedulla region. **(G)** Representative H&E staining images (left) and the relative polarized-light images (right) of the defect area. Data in **(E)** and **(G)** were derived from an identical femur. Bar, 500 μm. Data were represented as means ± s.e.m. Ordinary one-way ANOVA analysis. *P < 0.05. **P < 0.01. ***P < 0.001. SWL, standard white light; PWL, polarized white light.

After 7 days, SHAM mice and 5 of 12 OVX mice from each group were sacrificed, and the long bones were subjected to radiographic evaluation. The total BMD including trabecular and cortical bone revealed significant osteoporosis of the left tibiae in OVX mice compared to that of the sham-operated mice, analyzed by pQCT (Supplementary Figure S1B). The X-ray images showed that the defect region in the IDG-SW3 group was filled with mineralized callus, whereas the mineralized callus in the BMSC group or cell-free group was relatively smaller (Figures 5A,B). Then, these femurs were both subjected to paraffin embedding and sectioning. TRAP staining showed that the osteoclast surface per bone surface was increased in the defect region of the IDG-SW3 group compared to that in the other groups (Figures 5C,D).

Fourteen days after the drill-hole surgery, the μ-CT images showed that the cortical bone defect sites were almost bridged (Figure 5E); this was even observed in the cell-free group. However, the new bone in the intramedulla region was nearly diminished in the IDG-SW3 group, whereas in the hydrogel group or BMSC group, the intramedulla region still had a visible mineralized callus (Figure 5E). Consistently, statistical analysis revealed a lowest bone volume per tissue volume (BV/TV) of the intramedulla region in the IDG-SW3 group (Figure 5F), indicating that the mineralized callus was completely remodeled into a tube-like bone in the IDG-SW3 group. This result was further confirmed under the polarized light microscope observation, which showed that the tube-like bone was mostly lamellar bone in the IDG-SW3 group, whereas most of the tube-like bone in the BMSC group or hydrogel group remained woven bone (Figure 5G). Therefore, these results demonstrated that the IDG-SW3 cells could more rapidly repair the bone defects than BMSCs.

## Discussion

Bone defect repair is not a single bone formation process but a spatiotemporal change that is produced through a series of events, including immune response, bone formation, bone mineralization, and bone remodeling. Throughout the bone healing process, different stages involve specific biological functions of different cells and are precisely carried out in an ordered manner (Einhorn, 2005). IDG-SW3 cells are immortal murine osteoblast cells that can either be induced to self-renew or to differentiate into mature osteocytes, depending on the different culture medium (Woo et al., 2011). By culturing them with normal osteogenic condition, the IDG-SW3 cells will differentiate into osteocytes without proliferation (Woo et al., 2011). Importantly, the differentiation process perfectly matched the *in vivo* maturation of osteoblasts to osteocytes, including morphological and proteomic changes in preosteocytes, middle osteocytes, and late osteocytes. Currently, BMSCs are mostly used in the treatment of bone defects in animal models due to their self-renewal capability and osteogenic potential (Zong et al., 2010; Koob et al., 2011). It has been reported in the literature that scaffolds containing IDG-SW3 cells can promote the repair of skull bone defects in mice (Woo et al., 2011). Here, we compared the difference between IDG-SW3 and BMSCs in promoting bone defect repair.

The type I collagen is the most abundant matrix component in osteoid matrix (Engler et al., 2006). It has been found that the arrangement of type I collagen and its sensitivity to MMPs digestion influences the osteocytogenesis (Zhao et al., 2000). Matrigel contains a variety of extracellular matrix components, including various growth factors. It is known that cytokines such as FGF-2 are essential for osteocytogenesis *in vivo* and is important to bone repair process (Fei et al., 2013). In addition, the loose collagen hydrogel is more suitable for osteocytic process extension and process-network formation (Kurata et al., 2006). Thus, we used type I collagen and matrigel fusion gel. In contract with the pure type I collagen hydrogel, although it mimics the composition and the structure of native osteoid tissue, it is not favorable to cellular process extension and is more suitable for studying the spatially polarized process of osteocytogenesis *in vitro* (Robin et al., 2016). Of note, there are many other collagen-free 3D gels utilized in osteocyte culture systems, including calcium phosphate particles (Boukhechba et al., 2009), commercialized 3D polystyrene scaffolds (Spatz et al., 2015), and nanofiber-enabled layer-by-layer cell assembly (Yang et al., 2009), to mimic the structural complexity of osteoid, in which the osteocytes are completely embedded in the 3D matrix environment.

The results of Osteocalcin-Cre-driven DMP1 conditional knockout mice revealed that the mineralized matrix vesicle secretion mainly occurred on the membrane of cells in the lacunar–canaliculi network (Feng et al., 2006). Therefore, the extensive lacunar–canaliculi network and the suitable DMP-1 expression by IDG-SW3 cells in the type I collagen gel culture system both contribute to the surrounding matrix mineralization. The calcium-phosphorus content in the collagen gel mixed with IDG-SW3 cells was much enriched than that of BMSCs cells, although the mineralization products of these two cells are identical. These might be related to the insufficient DMP-1 expression and lacunar–canaliculi network in BMSCs.

The osteocyte and osteoclast co-culture system we used was similar to the study of Kurata et al. (2006) by directly plating the monocytes on the surface of the hydrogel containing osteocytes to study the osteoclastogenesis event alone. The difference is that the IDG-SW3 cell we used has a stronger mineralization ability than the MLO-Y4 cell line. Of note, the other *in vitro* studies prefer to plate osteocytes and osteoclasts on either side of an inert membrane with a pore size of less than 4 μm, which only permit the cellular process interaction (Honma et al., 2013; Skottke et al., 2019). This co-culture system is suitable for the cell collection and needs to be studied separately, and it is also convenient to study the molecules trafficking between them.

During the bone repair process, the mineralized callus needs to be shaped through a bone remodeling process, which is mainly based on the absorption of new bone by osteoclasts (Kenkre and Bassett, 2018). We found that TRAP activity was higher in the osteoclasts co-cultured with gels containing IDG-SW3 cells after 4 weeks of differentiation *in vitro*. This may be due to two reasons; our results showed that compared to BMSCs, the gel containing IDG-SW3 cells had more RANKL protein to promote osteoclast activation *in vitro*; in addition, studies have reported that osteoclasts are more fascinated in hydroxyapatite-coated surfaces (Charles and Aliprantis, 2014). The gel with IDG-SW3 cells contained a large number of mineralized particles, which may be the one reason for osteoclast activation. These results explained the broad TRAP-positive area in the mineralized callus of the IDG-SW3 group, which show that bone remodeling in the IDG-SW3 group was superior to that of the BMSC group.

Obviously, our work is still very preliminary to be clinically relevant. We found that the IDG-SW3 cells have stronger osteogenic capacity than BMSCs both *in vitro* and in osteoporotic bone healing, but this does not mean that we deny the value of BMSCs as a cell source for bone healing, because the functions of BMSCs in inflammation and hematoma formation were still unclear and isolating/expanding osteoblasts *in vitro* is difficult. However, the results in our study showed that the DMP-1 proteins expressed in BMSC-differentiated osteocyte are much fewer compared to that in IDG-SW3 cells, indicating that the BMSC-derived osteocytes *in vitro* are somehow different with the primary osteocytes. Thus, the point is to how to induce BMSCs differentiating to the mature osteocytes with physiological functions more effectively. Several researches reported that the physiological triggers (e.g., matrix mineralization, low oxygen tension, and mechanical stress) and the identified exogenous supplemental molecules (e.g., FGF-2, retinoic acid) could provide more interventions and options to modulate osteocytogenesis (Chen et al., 2018). Perhaps one day the mechanism of osteocytogenesis is uncovered, by then we could promote osteoblast-to-osteocyte transition to improve the therapeutic effect of BMSCs in bone defect repair.

## Data Availability Statement

All datasets generated for this study are included in the article/Supplementary Material.

## Ethics Statement

The animal study was reviewed and approved by the Shanghai Jiao Tong University Animal Care and Use Committee.

## Author Contributions

KC, QZ, and HK: cellular experiments. KC and QZ: physical and chemical properties analysis. KC and HK: animal experiment. NQ: radiographic examination and analysis. CL and FW: histological sections preparation and analysis. KY and YY: manuscript writing. KC, QZ, and KY: collection and/or assembly of data. KY, LD, and JQ: conception and design, financial support, manuscript writing, and final approval of manuscript. All authors contributed to the article and approved the submitted version.

## Conflict of Interest

The authors declare that the research was conducted in the absence of any commercial or financial relationships that could be construed as a potential conflict of interest. The reviewer KL declared a shared affiliation, with no collaboration, with the authors to the handling editor.

## Supplementary Material

FIGURE S1 **(A)** Schematic representation of the drill-hole defect model using micro-CT analysis. **(B)** After drill-hole surgery for 1 week, mice were sacrificed and the tibiae were subjected to pQCT examination. Total BMD (including trabecular and cortical bone) from tibiae of SHAM (*n* = 4) and OVX (hydrogel group, *n* = 5) mice. Data were represented as means ± s.e.m.

TABLE S1 Primers used in RT-PCR assay.

## Abbreviations

ALP: alkaline phosphatase
BMSCs: bone marrow-derived mesenchymal stem cells
DMP-1: dentin matrix protein 1
EDX: electron dispersive X-ray
FGF23: fibroblast growth factor 23
FTIR: Fourier transform infrared spectra
HA: hydroxyapatite
OC: osteocalcin
OVX: ovariectomized
RANKL: receptor activator of nuclear factor- κ B ligand
TRAP: tartrate-resistant acid phosphatase
XRD: X-ray diffraction.
