# Supplementary material for: High Mineralization Capacity of IDG-SW3 Cells in 3D Collagen Hydrogel for Bone Healing in Estrogen-Deficient Mice
Source: Front Bioeng Biotechnol. 2020 Aug 31;8:864. doi: 10.3389/fbioe.2020.00864 (PMC7488085; doi:10.3389/fbioe.2020.00864)
Supplement: FIGURE S1 — (A) Schematic representation of the drill-hole defect model using micro-CT analysis. (B) After drill-hole surgery for 1 week, mice were sacrificed and the tibiae were subjected to pQCT examination. Total BMD (including trabecular and cortical bone) from tibiae of SHAM (n = 4) and OVX (hydrogel group, n = 5) mice. Data were represented as means ± s.e.m. [file Image_1.pdf]

Supplementary Figure 1

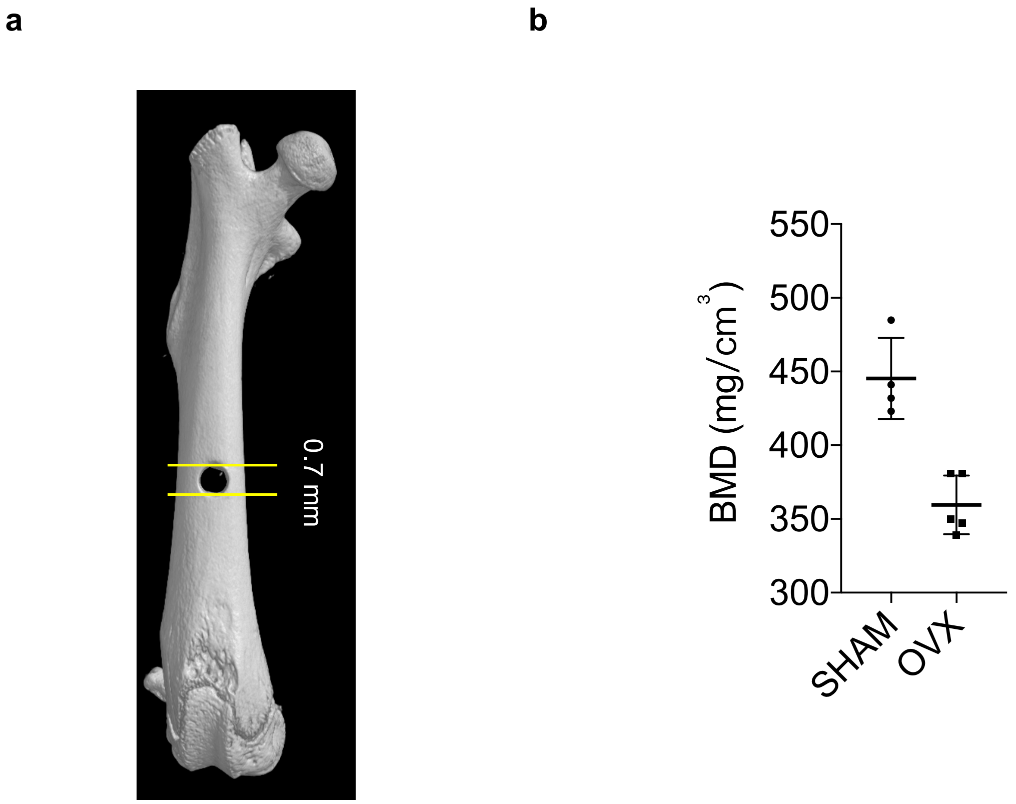

Supplementary Fig.1. (a) Schematic representation of the drill-hole defect model using micro-CT analysis. (b) After drill-hole surgery for 1 week, mice were sacrificed and the tibiae were subjected to pQCT examination. Total BMD (including trabecular and cortical bone) from tibiae of SHAM (n = 4) and OVX (hydrogel group, n = 5) mice. Data were represented as means ± s.e.m.
