# Supplementary material for: High Mineralization Capacity of IDG-SW3 Cells in 3D Collagen Hydrogel for Bone Healing in Estrogen-Deficient Mice
Source: Front Bioeng Biotechnol. 2020 Aug 31;8:864. doi: 10.3389/fbioe.2020.00864 (PMC7488085; doi:10.3389/fbioe.2020.00864)
Supplement: TABLE S1 — Primers used in RT-PCR assay. [file Table_1.DOC]

**Supplementary Table 1.** Primers used in RT-PCR assay.

| Names | Sequences (5’- 3’) |
| --- | --- |
| *Alp*-for | CTCCAAAAGCTCAACACCAATG |
| *Alp*-rev | ATTTGTCCATCTCCAGCCG |
| *Oc*-for | CACCTAGCAGACACCATGAG |
| *Oc*-rev | GTTCACTACCTTATTGCCCTCC |
| *Pdpn*-for | GGAGGGCTTAATGAATCTACTGG |
| *Pdpn*-rev | GGTTGTACTCTCGTGTTCTCTG |
| *Dmp1*-for | CCCAGTTGCCAGATACCAC |
| *Dmp1*-rev | CACTATTTGCCTGTCCCTCTG |
| *Sost*-for | ACAACCAGACCATGAACCG |
| *Sost*-rev | CAGGAAGCGGGTGTAGTG |
| *Fgf23*-for | GGTGATAACAGGAGCCATGAC |
| *Fgf23*-rev | TGCTTCTGCGACAAGTAGAC |
| *β-actin*-for | ACCTTCTACAATGAGCTGCG |
| *β-actin*-rev | CTGGATGGCTACGTACATGG |
